# Removal of psychopharmaceuticals from WWTP effluent by an algae–mussel trophic cascade: a potential nature-based solution?†

**DOI:** 10.1039/d5ew00011d

**Published:** 2025-05-09

**Authors:** Charlie J. E. Davey, Tom V. van der Meer, Thomas L. ter Laak, Piet F. M. Verdonschot, Michiel H. S. Kraak

**Affiliations:** a Institute for Biodiversity and Ecosystem Dynamics, University of Amsterdam Amsterdam The Netherlands c.j.e.davey@uva.nl; b Wageningen Environmental Research Wageningen UR P.O. Box 47 6700 AA Wageningen The Netherlands; c KWR Water Research Institute Nieuwegein The Netherlands

## Abstract

Psychopharmaceuticals are an emerging group of hazardous contaminants that pose a risk to the aquatic environment. Yet, modern wastewater treatment plants (WWTPs) do not remove them sufficiently to alleviate these risks. The present study aimed therefore to explore the effectiveness of an alternative nature-based tertiary treatment of WWTP effluent to remove psychopharmaceuticals. To this end, an algae–mussel trophic cascade setup was designed in which algae were grown in effluent over the course of 11 days and subsequently fed to mussels for a further 3 days. Removal of 30 psychopharmaceuticals for each of the treatments (algae, mussels, algae + mussels) was calculated relative to control samples, and removal efficiency was contextualised by performing an indicative risk assessment. Twelve psychopharmaceuticals were quantified during the experiment, with 11 encountered in all treatments. The compounds fell into 3 categories: positive removal (citalopram, lamotrigine, and venlafaxine), negative removal (carbamazepine, gabapentin, and pregabalin), and no significant changes in concentration (amitriptyline, quetiapine, tramadol, fluvoxamine, lidocaine, and ibuprofen). Both positive and negative removals were largely driven by the presence of the algae rather than that of the mussels. Compounds with a low p*K*_a_ showed negative removal due to the algal growth induced rise in pH, which was not negated by the mussels at the end of the cascade. Ibuprofen was not removed by any treatment and was also the only compound that represented a substantial risk. The cumulative risks indicated that the algal–mussel cascade actually increased the risk due to the negative removal of compounds present in high concentrations such as carbamazepine. Pregabalin and gabapentin also increased in risk, but did, however, not significantly change the overall risk from the analysed compounds due to their low concentrations. Since the presently designed nature-based treatment could not negate risk, it is not suitable for the removal of psychopharmaceuticals.

## Abstract 2

Psychopharmaceuticals are an emerging group of hazardous contaminants that pose a risk to the aquatic environment.

## Graphical abstract

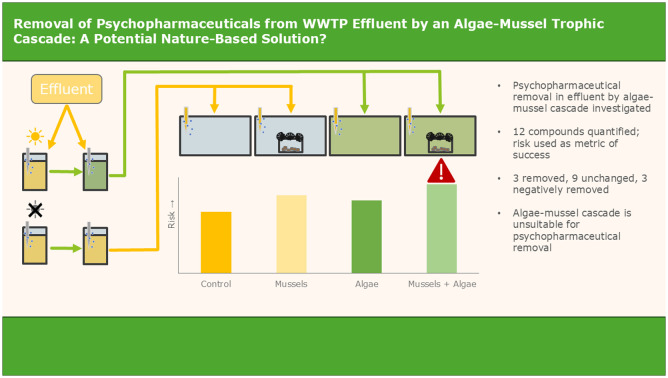

**Water impact**

A nature-based solution that utilises an algae–mussel cascade to remove psychopharmaceuticals from effluent was designed as an alternative to advanced treatments. Risk-based removal was used as a metric for success, and results show that the cascade did not reduce the risks due to pH changes from algal growth interfering with natural adsorption processes, rendering it unsuitable for psychopharmaceutical remediation.

## 1. Introduction

Psychopharmaceuticals are a unique category of emerging micropollutants, as they are designed to alter the neurochemistry of the human brain.^1,2^ Due to the similarities between the nervous systems of humans and other animals,^3,4^ these drugs can also act on the nervous systems of non-target species, leading to changed species interactions, which may ultimately affect aquatic ecosystem functioning.^5–12^ Psychopharmaceuticals include antidepressants, anxiolytics, antipsychotics, and illicit drugs, amongst others.^6^ These substances are commonly and increasingly used in modern society, but poorly removed from wastewater by conventional treatment steps at WWTPs^13–16^ leading to their frequent detection and increasing concentrations in WWTP effluent and receiving water bodies.^13–15,17–25^ Due to this insufficient removal, legislative concentration-based and risk-based targets are not being met.^15,16,26^

To enhance the removal of psychopharmaceuticals from wastewater, several advanced treatment options have been explored and proven efficient.^27–29^ Therefore, such advanced treatments are currently considered to be a necessity in many developed economies.^29^ Yet, the inclusion of advanced treatment processes, such as membranes, activated carbon, ozonation, and other advanced oxidation processes significantly increases the capital, operation, and maintenance costs of WWTPs by a factor of around 1.5–2.1,^30^ which severely limits the options available for developing regions. Additionally, high energy consumption and CO_2_ emissions could put advanced treatment implementation at odds with other sustainability goals. Therefore, it is imperative to explore alternative and cost-effective treatment options that can achieve efficient psychopharmaceutical removal while minimizing the financial costs involved, allowing a global application.

Nature-based solutions (NBSs) have emerged as a potential alternative to tertiary advanced treatments in modern WWTPs, and as an alternative for secondary treatments in older or non-existent WWTPs, offering numerous advantages in terms of efficiency, sustainability, and cost-effectiveness.^31^ NBSs involve the utilisation of natural systems, such as constructed wetlands^32,33^ or stabilisation ponds,^34^ amongst others^31^ to polish WWTP effluent. These systems harness the inherent capabilities of plants, animals, microbes, and natural materials to remove contaminants through various physical, chemical, and biological mechanisms. NBSs have a range of additional advantages depending on the type of treatment implemented. These advantages can range from mimicking natural ecosystems in the case of constructed wetlands, which in turn promotes biodiversity by habitat creation, to acting as a carbon sink *via* the production of biomass.^31^ Consequently, the interest in NBS by stakeholders, including the European Commission, has been rising over recent years.^35^ However, NBSs encounter frequent challenges, including higher land use compared to conventional treatment, unstandardised designs, and regular maintenance including vegetation maintenance and sediment/sludge removal.^31^

To circumvent some of the common challenges of larger NBSs such as constructed wetlands, a more controlled and concise use of algae has been proposed. Algae have been used to remove nutrients^36^ and to provide oxygen for biotic and abiotic degradation processes,^37,38^ as well as a substrate for adsorption and subsequent sedimentation of micropollutants^39^ and heavy metals,^40^ albeit with some varying results.^37,39^ However, the removal of sedimented algae can be a costly maintenance process, especially at larger scales.^41^ Yet, it is possible to reduce the algal disposal costs by employing filter-feeders, such as mussels, to consume the algae.^36,39^ Hence, an algal–mussel cascade could provide micropollutant removal at low costs, serving as a potential nature-based alternative to advanced effluent treatment.

Therefore, the present study aimed to design and test the effectiveness of a nature-based treatment to remove psychopharmaceuticals from WWTP effluent. The setup consisted of an algae–mussel trophic cascade in which algae were grown in effluent and subsequently fed to mussels. Comparing the concentrations of psychopharmaceuticals in the wastewater effluent at various stages and in control samples allowed the estimation of the removal efficiency of psychopharmaceuticals using this low-cost, low-tech NBS. We contextualised the obtained removal efficiencies by calculating the ecotoxicological risk of the remaining psychopharmaceutical concentrations in the wastewater^16^ by comparing these to available ecotoxicity data.

## 2. Materials & methods

### 2.1 Materials

A 200 L aliquot of secondary treated effluent was collected from the WWTP of Rhenen (46 000 population equivalents, Remmerden, The Netherlands (51°58′27.6′′N 5°31′55.6′′E)), which was a conventional University of Cape Town (UCT) carrousel with an additional filtration step (100 μm). The selected psychopharmaceuticals for LCMS analysis consisted of 30 compounds (ESI S1†). Labelled and unlabelled standards were purchased from Sigma-Aldrich Chemie (Schnelldorf, Germany). Stock solutions were prepared using MeOH and stored at −20 °C. Oasis HLB cartridges (6 cc, 150 mg) were purchased from Waters (Etten-Leur, the Netherlands). The solvents used for solid phase extraction (SPE), chromatographic separation and stock solutions were of LC-MS grade, obtained from Biosolve (Valkenswaard, the Netherlands). Ultra-pure water used for SPE and separation was produced by a Milli-Q® Direct Water Purification System from Merck (Damstadt, Germany).

### 2.2 Algae–mussel trophic cascade

**Fig. 1:**
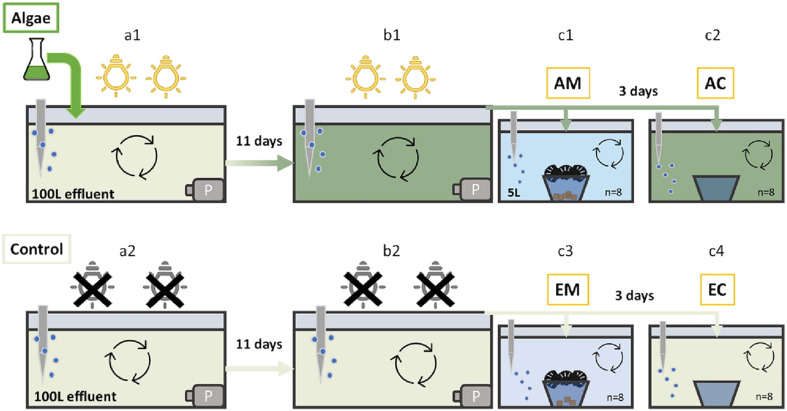
Schematic overview of the experimental setup (taken from van der Meer *et al.*)^36^ Algae grown in effluent (a1) and control effluent without algae (a2). End of the 11-day algae growth period (b1 and b2). Mussel filtration phase: effluent with algae and mussels (c1, algae–mussel, AM), effluent with algae but without mussels (c2, algae–control, AC), effluent with mussels, but without algae (c3, effluent–mussels EM), and effluent without mussels and without algae (c4, effluent–control, EC). P = aquarium pump.

A detailed outline of the algae–mussel cascade was published by van der Meer *et al.*,^36^ as shown in Fig. 1. In brief, the effluent was transported to the laboratory, where two 100 L aquaria were filled. Algae (*Tetradesmus obliquus*) were added to one aquarium under a light–dark cycle of 16 h : 8 h, while the other served as a control and was kept in the dark by covering it in aluminium foil, preventing native algal growth (ESI S8†). After 11 days of algal growth, either 5 L of the algal effluent or 5 L of the control effluent was poured into 16 glass tanks each. Mussels (*Dreisenna bugensis*) were added to 8 of the algae tanks and 8 of the control tanks, resulting in a full factorial 2 × 2 design with 8 replicates per treatment, consisting of: 1] effluent with algae and mussels (AM); 2] effluent with algae but without mussels (algae control, AC); 3] effluent with mussels but without algae (EM); and 4] effluent control (EC) without algae and without mussels. This setup allowed for the calculation of psychopharmaceutical removal by algae and mussels separately, as well as together, by comparing each treatment with the control.

The mussels were placed on a metal mesh suspended over a glass cup to maximise the mussel filtration efficiency. To control for glass adsorption of the compounds, glass cups were also added to the treatments without mussels. The mussels were allowed to filter for 3 days with intermittent stirring to re-suspend settled algae outside of the cup. Water samples of approximately 1 L were collected at each step of the experiment and stored at −20 °C until further analysis. This included the starting effluent (Fig. 1a1 and a2), the effluent in the algae tank (Fig. 1b1) and control tank (Fig. 1b2) after 11 days, as well as samples of each experimental replicate after mussel filtration of 3 days (Fig. 1c1–c4).

### 2.3 Solid phase extraction of water samples

Sample extraction was performed according to Davey *et al.*^16^ Briefly, after thawing, samples were spiked with labelled standards and shaken at 90 rpm for 30 minutes. Outlets, tubes, and adapters were cleaned with ultrapure water followed by methanol. Conditioning of the Oasis HLB cartridges (150 mg, 6 cc) was done with 6 mL MeOH and 6 mL ultrapure water. After sample loading (50 mL, in duplicate), the cartridges were dried for 30 minutes under vacuum and washed with 6 mL of ultrapure water. Before elution, a 0.22 μm polypropylene syringe filter was placed between the cartridges and SPE inlets. Elution was achieved with 2 × 5 mL methanol under vacuum. The collected elution fractions were evaporated under a gentle nitrogen flow at 37 °C to <1 mL and reconstituted to 1 mL in methanol and subsequently stored (−20 °C) until analysis.

### 2.4 UHPLC-HRMS analysis of psychopharmaceuticals

Psychopharmaceuticals were analysed according to Davey *et al.*^16^ Briefly, a LC system (Nexera 30 Schimadzu, Den Bosch, The Netherlands) was coupled to a maXis 4G quadrupole time-of-flight HRMS (qToF/HRMS) upgraded with a HD collision cell and ESI source (Bruker Daltonics, Leiderdorp, The Netherlands). The LC column used was an Acquity UPLC CSH C18 column (130 Å, 2.1 × 150 mm, 1.7 μm, Waters Corporation, Milford) kept at a temperature of 40 °C. The mobile phases consisted of ultrapure water (Milli-Q) with 0.05% acetic acid (A) and MeOH (B). The gradient started with a 7-minute equilibration at 10% B and gradually increased to 100% B in 10 minutes, held at 100% B for 5 minutes, and then brought back to 10% B in 0.5 minutes, totalling 22.5 minutes. The flow rate was 0.3 mL min^−1^ and the injection volume was 20 μL. The samples were analysed in both positive and negative modes acquiring HRMS1 spectra for 20–1000 *m*/*z* with a resolving power of 30 000–60 000 at full width half maximum (FWHM), with a spray voltage of +3.5 kV and −3.5 kV for positive and negative modes respectively.

Both qualification and quantification of target compounds were carried out with TASQ version 2021.0 316 (Bruker Daltonics, Leiderdorp, the Netherlands). Qualification was based on the mass accuracy of full-scan HRMS spectra and MS/MS ions acquired in data-independent MS/MS mode (DIA), and on their retention time match with the calibration series.

During quantification, only the chromatograms with a retention time (RT) of >1 minute, RT tolerance of ±0.3 minutes, mass tolerance of 0.002 Da, detectable qualifier ion, mSigma of <100, and a peak intensity of >1000 were considered (ESI S1†). Calibration curves for quantification were obtained by analysing ultrapure water spiked with target compounds, in serial dilutions to obtain 18 concentrations. The 18 calibration solutions were further spiked to contain 10 μg L^−1^ of internal standard. Internal standards were also added to the samples to adjust for losses during SPE, which were calculated automatically in TASQ.

### 2.5 Removal efficiency, risk calculation, & statistics

Data analysis was carried out using Microsoft Excel. Procedural duplicates were averaged, blanks were subtracted, and data were corrected for dilution factors to obtain the concentrations of the pharmaceuticals in the samples. For each psychopharmaceutical, differences in concentration between starting aquaria (*n* = 2 tanks at the start of the experiment), EC, EM, AC, and AM samples (all *n* = 8) were tested using a non-parametric Kruskal–Wallis test followed by Dunn's test (ESI S6†) using the Real Statistics Resource Pack Excel add-in (version 8.9.1).^42^ Subsequently, removal of the psychopharmaceuticals by the different biological treatments was calculated by comparing the concentrations of the psychopharmaceuticals in the AM, AC, and EM treatments to those in the control samples (EC). Only the treatments that were statistically significantly different from the control (EC) were considered as having removed the pharmaceutical.

To contextualise the removal efficiencies of the psychopharmaceuticals by the organisms, an indicative risk assessment was performed. To this end predicted no effect concentrations (PNECs) for freshwater were obtained from the NORMAN database^43^ (ESI S2†). The concentrations of the psychopharmaceuticals in each sample were divided by the corresponding PNECs to calculate the risk quotients (RQs) per compound, which were then plotted as boxplots. Cumulative risk was calculated by summing the RQs of all compounds per treatment.

Pearson correlations were performed on the removal results to evaluate which physico-chemical parameters can explain the observed removals (ESI S7†). Since biodegradation and adsorption were expected to play the main role in removals, these parameters were selected for the correlations. To this end biodegradation half-lives were predicted using Episuite's BCFBAF module,^44^ while p*K*_a_ values were obtained from PubChem,^45^ and solubility data at different pH levels were calculated using Chemicalize.^46^ Ionisation was calculated using the measured pH during algal cultivation and the p*K*_a_ for each pharmaceutical was calculated using the following formula (ESI S9†):1
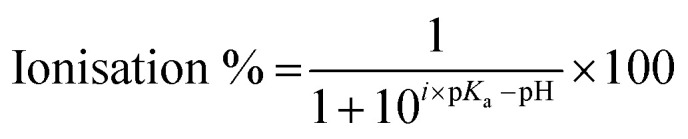
where *i* is the ionisation type, 1 for acidic and −1 for basic, p*K*_a_ is the acid dissociation constant for the individual compound, and pH is the negative log of the proton concentration in solution.

## 3. Results

### 3.1 Quantification of psychopharmaceuticals & removal percentages

A total of 27 out of the 30 compounds could be analysed and qualified (ESI S3†). Caffeine and clozapine were excluded from further analysis due to contamination issues and phenacetin was removed due to poor calibration. One sample (EC-h) had a bad injection, and was thus removed from the study, leaving 7 replicates for the EC results (ESI S6†). Twelve compounds returned concentrations above the limit of quantification in at least one sample. Amitriptyline only returned concentrations in the starting samples, and was therefore excluded from the table on removal percentages (Table 1, ESI S4†), leaving 11 compounds that returned results in all treatments (Table 1).

**Table 1:** Mean removal (±sd, EC: *n* = 7, others: *n* = 8) defined as the relative difference in concentration compared to the control (EC) samples for the 11 compounds that returned results in all sample types. Kruskal–Wallis significance indicates if there was a significant difference between the treatments

Psychopharmaceutical	Algae (%)	Mussels (%)	Algae & mussels (%)	Kruskal–Wallis significance
Citalopram	78.4 ± 47	41.7 ± 37	95.4 ± 28	Yes
Lamotrigine	77.4 ± 18	−40.1 ± 16	41.1 ± 29	Yes
Venlafaxine	25.6 ± 22	−7.6 ± 19	27.4 ± 28	Yes
Quetiapine	−34.9 ± 29	−14.9 ± 35	4.6 ± 30	No
Tramadol	9.7 ± 25	−13 ± 20	18.8 ± 35	Yes
Fluvoxamine	−0.1 ± 20	−19.9 ± 11	−24.7 ± 21	Yes
Lidocaine	−5 ± 12	−8.5 ± 13	0.2 ± 19	No
Ibuprofen	−4.5 ± 23	7.8 ± 31	2.2 ± 31	No
Carbamazepine	−78.9 ± 30	−52.2 ± 20	−132.4 ± 40	Yes
Pregabalin	−469.6 ± 36	−222.3 ± 36	−448.6 ± 34	Yes
Gabapentin	−598.7 ± 25	−299.2 ± 14	−572.5 ± 14	Yes

Removal percentages ranged from −600% to 95%, with three compounds (citalopram, lamotrigine, and venlafaxine) exhibiting statistically significant removal by algae, mussels, or both (KW *χ*^2^ = 23.2–29.9, df = 5, all *p*s < 0.05). Two compounds (quetiapine and tramadol) showed significant decreases relative to the start (KW *χ*^2^ = 5.5–16.0, df = 5, all *p*s < 0.05), but no difference between treatments and the control, fluvoxamine showed a significant increase relative to the start (KW *χ*^2^ = 19.0, df = 5, *p* < 0.05), while another two compounds (ibuprofen and lidocaine) showed no significant changes in concentrations (KW *χ*^2^ = 5.0–6.1, df = 5, all *p*s = 0.3–0.31). Three compounds (carbamazepine, pregabalin, and gabapentin) showed statistically significant negative removal in the algae, mussels, or both (KW *χ*^2^ = 18.9–24.0, df = 5, all *p*s < 0.05).

### 3.2 Concentrations of psychopharmaceuticals & corresponding indicative risks

**Fig. 2:**
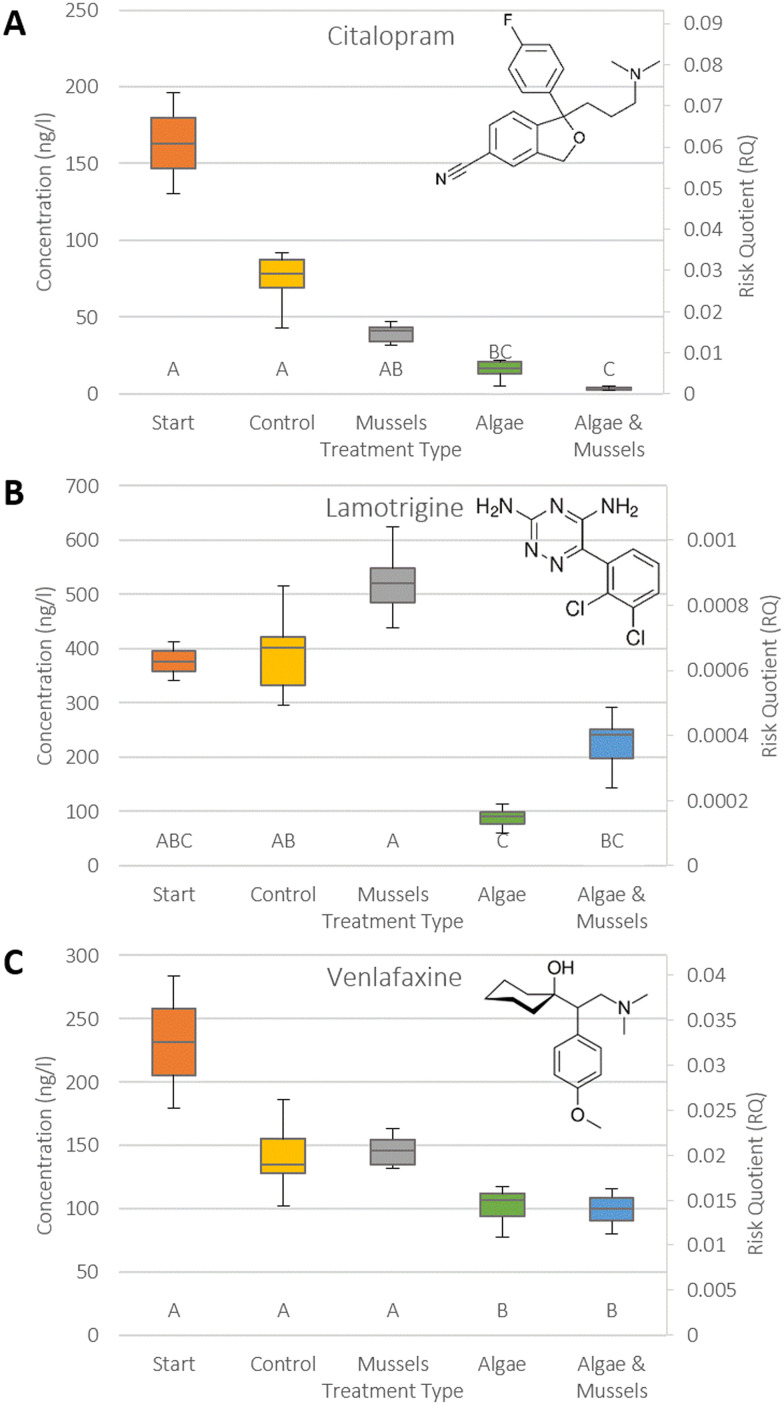
Combination boxplots showing both concentration and risk in each sample type for the three psychopharmaceuticals that showed a significant positive removal in at least one of the samples: citalopram (A), lamotrigine (B) and venlafaxine (C). The start concentration is provided for context. An RQ > 1 indicates a risk. Statistical significance categories as identified by Dunn's test are indicated by the letters on the bottom, where a matching letter indicates no significant difference between samples. Structural formulae for the compounds have also been provided. Whiskers represent upper and lower quartiles, while the box represents middle upper and middle lower. The central line indicates the median value (EC: *n* = 7, others: *n* = 8).

**Fig. 3:**
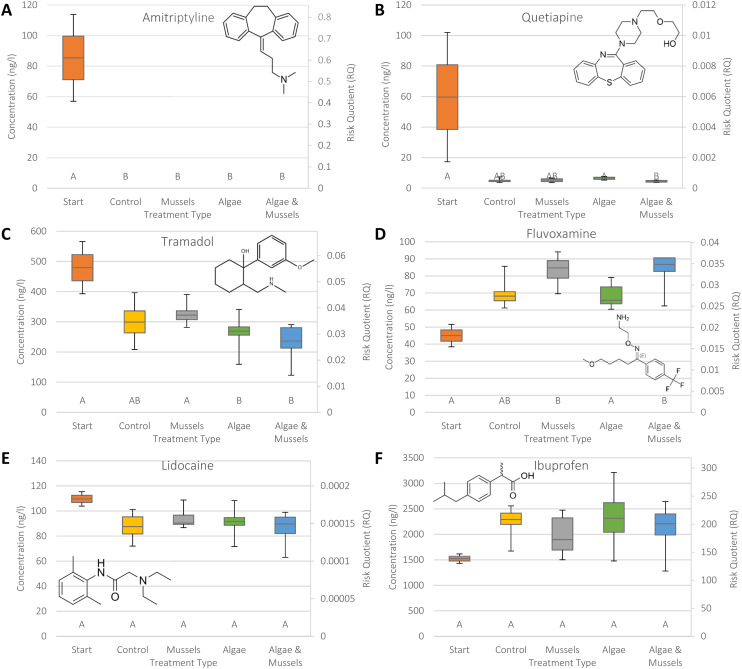
Combination boxplots showing both concentration and risk in each sample type for the six psychopharmaceuticals that showed no significant changes in concentration in any of the samples: amitriptyline (A), quetiapine (B), tramadol (C), fluvoxamine (D), lidocaine (E), and ibuprofen (F). The start concentration is provided for context. An RQ > 1 indicates a risk. Statistical significance categories as identified by Dunn's test are indicated by the letters on the bottom, where a matching letter indicates no significant difference between samples. Structural formulae for the compounds have also been provided. Whiskers represent upper and lower quartiles, while the box represents middle upper and middle lower. The central line indicates the median value (EC: *n* = 7, others: *n* = 8).

**Fig. 4:**
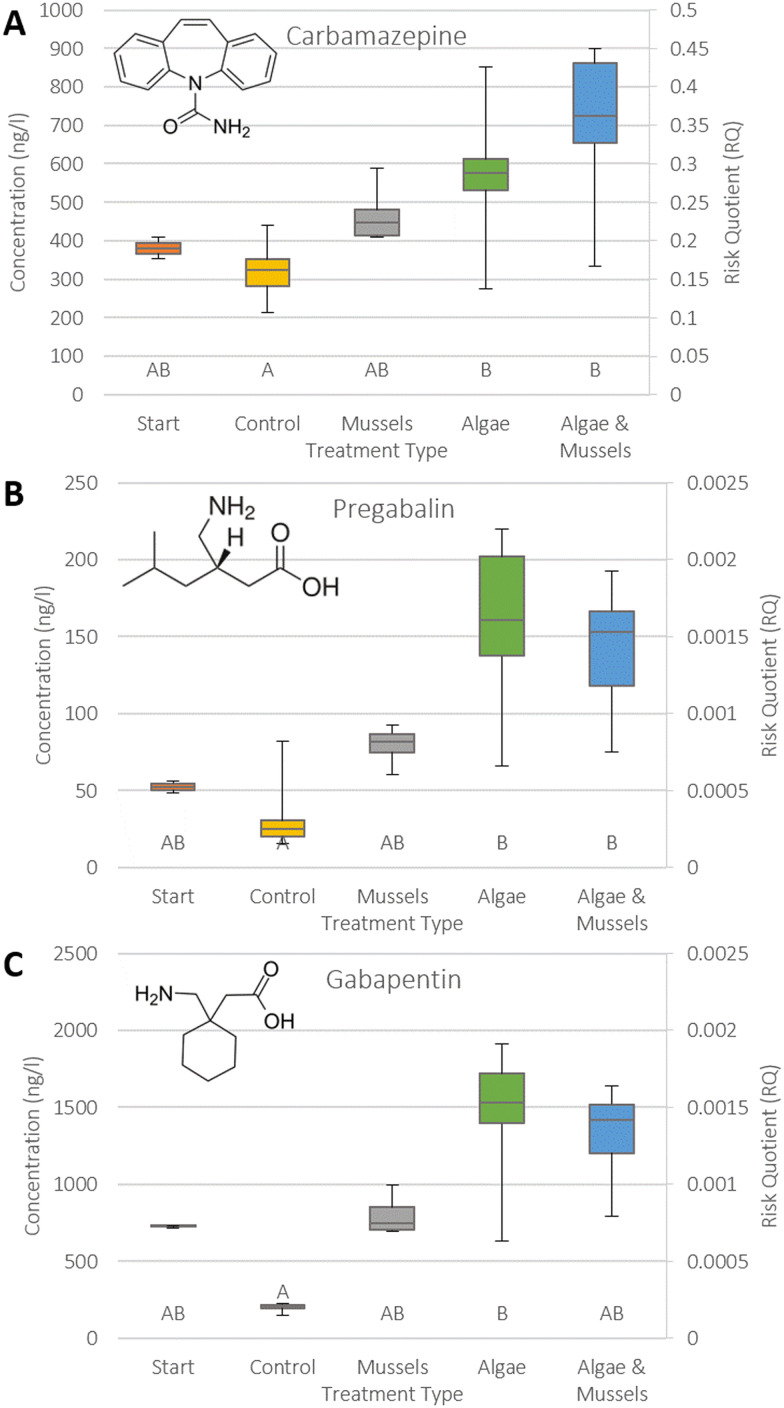
Combination boxplots showing both concentration and risk in each sample type for the three psychopharmaceuticals that showed a significant negative removal in at least one of the samples: carbamazepine (A), pregabalin (B), and gabapentin (C). The start concentration is provided for context. An RQ > 1 indicates a risk. Statistical significance categories as identified by Dunn's test are indicated by the letters on the bottom, where a matching letter indicates no significant difference between samples. Structural formulae for the compounds have also been provided. Whiskers represent upper and lower quartiles, while the box represents middle upper and middle lower. The central line indicates the median value (EC: *n* = 7, others: *n* = 8).

The 12 compounds were split into three categories: positive removal (Fig. 2), no significant changes in concentration (Fig. 3), and negative removal (Fig. 4). The results for risk showed that RQs were mostly well below 1 (ESI S5†), except for amitriptyline (Fig. 3A, median 0.61 in start), carbamazepine (Fig. 4A, median 0.36 in AM), and especially ibuprofen (Fig. 4F, RQ > 100 in all samples).

Positive removal of citalopram, lamotrigine, and venlafaxine was driven by algal growth, with mussels mostly playing a non-significant role. For lamotrigine (Fig. 2B), mussels appeared to hamper the removal by the algae, since the concentration in the AM sample was not significantly different from the control, while in the algae treatment it significantly differed from the control (ESI S6†). The RQs for these three compounds were well below 1 in both starting conditions and after all treatment types, demonstrating a lack of risk.

The compounds for which no significant changes in concentration were observed fell into two categories: the compounds that did not show significant differences between the four treatments, but their concentrations were significantly different compared to the starting concentrations (amitriptyline, quetiapine, tramadol, and fluvoxamine Fig. 3A–D), and the compounds that showed no significant changes in concentration over the entire experiment, including the start concentration (lidocaine and ibuprofen, Fig. 3E and F). The amitriptyline concentrations in the four treatments were all below the LOQ. For quetiapine, the concentration in the AM samples was significantly lower compared to the starting samples, but did not differ between the control and the other treatments. The tramadol and fluvoxamine concentrations also showed significant differences between the start and two treatments, lower in the case of tramadol, higher in the case of fluvoxamine, but again there were no differences between the treatments and the control. The RQ for amitriptyline was within 1 order of magnitude of 1, while the RQ of ibuprofen exceeded 1 by over 2 orders of magnitude, indicating a very high risk.

Negative removal was observed for carbamazepine, gabapentin, and pregabalin, and was mostly significant in the algae containing treatments (AC and AM). While none of the compounds presented a risk, the RQ for carbamazepine was within 1 order of magnitude of 1 in all sample types. It should also be highlighted that the negative removal caused a net gain in risk for the three compounds in this category.

### 3.3 Cumulative risk

**Fig. 5:**
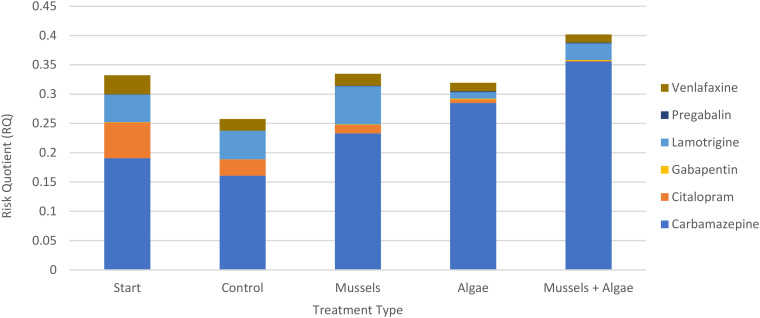
Cumulative risk (in median RQs) of compounds with statistically significant results (see Fig. 2 and 4) in each of the treatments.

By adding the risks of all compounds for which significant changes in concentration were observed, either positive or negative, the change in risk due to the treatments was +30%, +24%, and +56% for EM, AC, and EM respectively, indicating that there was a net gain in risk from psychopharmaceuticals due to the presence of both algae and mussels, as well as in combination compared to the control samples. This is largely due to the statistically significant negative removal of carbamazepine (Fig. 4a and 5). While the cumulative risk decreased between the start and the control, the addition of mussels or algae appeared to negate the reduction in cumulative risk over time, while the combination of mussels and algae produced a higher risk than both the start and the control samples. When all compounds are considered together, the cumulative risk is identical to the risk of ibuprofen, because this was several orders of magnitude higher than for the other compounds. As the ibuprofen concentrations remained unchanged, so did the corresponding risk (ESI S7†).

### 3.4 Impacts of pH changes from algae cultivation on the physicochemical properties

**Fig. 6:**
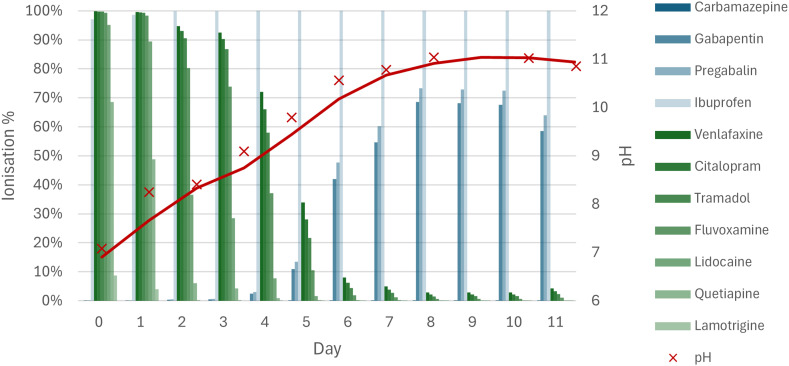
Rise in pH of the algae cultivation tank (red) and the ionisation % calculated from the p*K*_a_ for the compounds in Fig. 2–4. Amitriptyline is not shown as it fell below the LOQ in the control (Fig. 2). Compounds are listed in order of decreasing p*K*_a_ in the respective groupings of acidic (blue) and basic (green). Note that the pH measurement on day 9 was anomalous and removed from this figure (ESI S8†).

During algae cultivation, the pH of the algae tank rose from 6.7 to 11, while the pH in the control tank rose to 8.9 (ESI S8†). The p*K*_a_ of most selected psychopharmaceuticals falls within these pH ranges (ESI S7†), meaning that many psychopharmaceuticals have undergone a change in ionisation during the algal growth phase of the experiment (Fig. 6, ESI S9†). Broadly, acidic compounds became increasingly ionised (blue, Fig. 6) while basic compounds became neutralised with rising pH (green, Fig. 6). Carbamazepine, however, remained neutral and ibuprofen only changed marginally (from 97% to 100% ionised) during the change in pH in the algae tank.

**Fig. 7:**
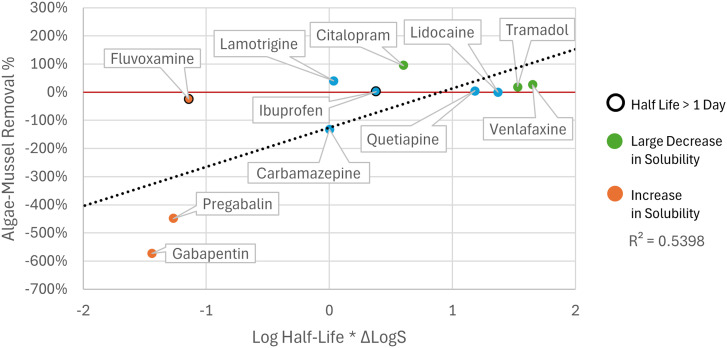
Removal in the AM treatment (*Y*-axis) and the log half-life multiplied by the change in log solubility between pH 7 and pH 11 (*X*-axis). Orange points indicate compounds increased in solubility during algal cultivation, while green points indicate compounds with a large (>−2) decrease in solubility. Points highlighted with a bold outline indicate compounds for which the biodegradation half-life was longer than one day.

The change in ionisation state led to a change in solubility of the psychopharmaceuticals, but the observed removal and change in aqueous solubility (Δlog *S*) did not correlate significantly (*r* = 0.6, *p* = 0.1). Yet, when the change in solubility was paired with biodegradability (expressed as log half-life), a significant (*r* = 0.73, *p* < 0.05) positive correlation was observed, indicating that both solubility and biodegradability played a role in explaining the differences in removal of the psychopharmaceuticals (Fig. 7, ESI S7†).

## 4. Discussion

### 4.1 Performance of the algae–mussel cascade

The results for the removal by the algae–mussel cascade were ambiguous, with three compounds showing positive removal, three compounds showing negative removal, and six compounds showing no significant changes in concentration. Differences in removal between different compounds is not uncommon for (psycho)pharmaceutical remediation.^47^ For the compounds that were removed by the setup, removal was driven by algal growth, rather than adsorption to mussels. Citalopram and lamotrigine showed better removal (95% in AM, 77% in AC, respectively) than some conventional advanced treatments in recent studies, where ozone was reported to remove 63% and 56%, respectively, while GAC removed 58% and 61%, respectively; however most results for pharmaceutical removal using conventional advanced treatments are better.^29,48–51^

The negative removals in combination with the compounds for which no changes in concentration were observed drove the lack of risk reduction, with the bulk of the risk coming from ibuprofen (no significant removal) and carbamazepine (negative removal), which gives an indication that this setup is unsuited to remove these psychopharmaceuticals, and more engineered setups such as ozone or active carbon may be required to mitigate the risk of these compounds.

The mussels effectively consumed the algae,^36^ and did not significantly re-release the psychopharmaceuticals back into the water, indicating that the mussels worked as intended. The mussels themselves did not drive any removal of psychopharmaceuticals. While literature on pharmaceutical removal by mussels is scarce, direct mussel removal of pharmaceuticals has been reported with ambiguous results.^39^

### 4.2 Negative removal of psychopharmaceuticals

While the control pH was around 7 during the mussel phase of the experiment (ESI S8†), the algal culturing of 11 days caused the pH to rise from 7 to 11, which is higher than in other algal setups reported in literature (*e.g.* 8.6 (ref. 52)). Consequently, the solubility of pregabalin and gabapentin increased significantly during the algal culturing, owing to the carboxylic group and a lower p*K*_a_ than the other compounds, resulting in negative removal (ESI S7†). Ibuprofen also contains a carboxylic group and showed negative removal in the algae-only treatment, albeit not significant. Algal cell walls also contain carboxylic groups, amongst others, which bind with ions and similar groups in micropollutants, which is the driving force for heavy metal^53^ and micropollutant removal.^38,54^ These interactions have been shown to be disrupted by changes in pH,^55^ since at high pH the carboxylic groups will become ionised both on the compound and on the cell wall, disrupting the hydrogen bonding between them and introducing electrostatic repulsion. The increase in concentration (*i.e.* negative removal) for some of the compounds thus suggests that at the start of the experiment they were mostly bound to suspended matter (abiotic organic particles, microorganisms, *etc.*), but were released again with the rise in pH, explaining the increase in concentration, and subsequent negative removal of these compounds. For carbamazepine, the p*K*_a_ did not correspond to a change in ionisation, thus remaining neutral throughout the pH swing. However, the pH swing could affect the sorption properties of the algal cell walls, amongst other biota and suspended particles, resulting in a release of carbamazepine into the water phase with increasing pH.^55^ Other explanations for the observed negative removal include product-to-parent transformation by deconjugation of metabolites.^56^ However, for compounds such as gabapentin, metabolites are not formed,^57^ while others do not have conjugated versions,^58^ indicating that this was not the case.

While other experimental algal removal studies adjusted the pH,^38^ the present study did not opt for this since this would not be in line with an NBS. Consequently, the present setup did not work for some of the psychopharmaceuticals. However, the use of substrates such as gravel or soil has been shown to buffer the pH in stabilisation ponds undergoing an algal bloom, without active pH adjustment.^59^ Moderating the pH *via* substrates or another method may lead to positive removals for the compounds that were not removed, or reported negative removal efficiencies,^60^ but this would need to be tested at scale.

### 4.3 Pros & cons of risk-based removal

To contextualise the removal of psychopharmaceuticals, the present study paired removal to ecotoxicity to derive indicative risks. While this has been used in recent studies,^16^ it has some limitations, especially regarding the PNECs extracted from the NORMAN database.^43^ The NORMAN PNECs contain a mixture of PNECs derived from both experimental data and modelled data, use different assessment factors, and do not state if acute or chronic data were used, or what the exact endpoints of the tests were. Missing ecotoxicity data is known to be a major limitation in risk assessment for psychopharmaceuticals,^6^ and therefore the RQs produced in this paper need to be met with appropriate scepticism. Nonetheless, the use of the PNECs allows performance of an indicative risk assessment to compare the (lack of) risk reduction within a single study.

The current study also did not quantify transformation products, as this would require (labelled) analytical standards of a spectrum of potential metabolites which are not easily available. There are legitimate reasons for studying transformation products, especially since these compounds may be found in higher concentrations than their parent compounds and be biologically active and thus pose a risk.^61–64^ A more comprehensive risk assessment should thus also include metabolites and transformation products from (psycho)pharmaceuticals, and factor in any potential mixture effects.^65–67^

### 4.4 Rhenen WWTP and demographics

The present study did not detect as many psychopharmaceuticals as anticipated, nor in as high concentrations as expected based on other recent studies in the Netherlands using the same analytical methodology.^16^ While it was expected that compounds such as carbamazepine would be detected in higher concentrations^16^ or a higher number of SSRI antidepressants would be detected in line with Dutch prescription data,^16,68^ this was not the case. Additionally, it was unexpected that ibuprofen returned very high concentrations compared to other recent Dutch studies.^16^ This is also despite the fact that the setup of the Rhenen WWTP is more rudimentary than more sizable WWTPs, such as the nearby Amsterdam West.^69^ This could be explained by demographic differences, since the inhabitants of Rhenen are proportionally older than those in Amsterdam,^70^ and younger people and teenagers proportionally use more psychopharmaceuticals,^71–74^ while older people are more likely to use analgesics.^75,76^ This demographic difference may explain the large differences in concentrations of Rhenen effluent when compared to other Dutch effluents.^16^

### 4.5 Future perspectives

The present study aimed to test the effectiveness of an algae–mussel cascade NBS in removing psychopharmaceuticals, specifically using risk-based removal as a metric for success. The present setup did, however, not achieve a reduction in risk, but an increase. The lack of any significant change in the ibuprofen concentration contributed the most to the overall risk, as ibuprofen carried the highest risk by multiple orders of magnitude, while carbamazepine contributed the most to the negative removal and increase in risk. The three compounds that did show positive removal and reductions in risk were not enough to negate the increase in risk caused by carbamazepine, nor made an impact on the risks from ibuprofen. Hence, the cascade did not remove psychopharmaceuticals and therefore did not negate the associated risks.

Nonetheless, potential benefits of the cascade were shown. The cascade also does not require any additional chemicals and could be a sizable CO_2_ sink at scale, bringing it in line with sustainable development goals and green chemistry principles.^77,78^ Furthermore, both mussels and algae have been shown to remove other micropollutants, such as heavy metals and nutrients.^36,38,39,53,54^ Further studies are needed into the use of algae and mussels for the purpose of pollutant removal, since these organisms show promise for other substances.^36,38,53,54^ It has been shown that different species of algae can remove micropollutants at different rates,^79^ which could indicate that there is room for improvement through organism selection. A different selection of mussels would impact the removal of both algae and micropollutants, since filtrations rates vary over species, and depend on environmental conditions such as phytoplankton concentration,^80,81^ salinity,^82^ and temperature,^81^ amongst other variables. In addition, designing a different nature-based bioreactor containing sediments and other organisms, might be able to buffer the rise of pH stemming from algal growth and activity. These potential changes to the cascade setup might also mitigate negative effects on removal of the tested psychopharmaceuticals, while keeping the setup in line with NBS goals and bringing the cascade more in line with constructed wetlands.^59,60^

## 5. Conclusions

The present NBS for psychopharmaceutical removal using an algal-mussel cascade did not result in a net reduction in risk. A pH increase due to algal growth was suspected to cause negative removals of some psychopharmaceuticals due to the changes in solubility, which resulted in an increase in risk. If the pH increase could be buffered by using substrates, then it may be possible to alleviate the negative removal, or even turn these into positive removal. The cumulative risks indicated that the algal–mussel cascade actually increased the risk due to the negative removal of certain psychopharmaceuticals. Since the presently designed nature-based treatment could not negate risk, it is not suitable for the removal of psychopharmaceuticals.

## Author contributions

Charlie J. E. Davey: conceptualization, methodology, validation, formal analysis, investigation, resources, data curation, writing – original draft, writing – review & editing, visualization, project administration. Tom V. van der Meer: conceptualization, methodology, investigation, writing – review & editing. Thomas L. ter Laak: validation, writing – review & editing, supervision. Piet F. M. Verdonschot: conceptualization, writing – review & editing, supervision. Michiel H. S. Kraak: conceptualization, writing – review & editing, supervision, funding acquisition.

## Conflicts of interest

The authors declare that they have no known competing financial interests or personal relationships that could have appeared to influence the work reported in this paper.

## Supplementary Material

EW-011-D5EW00011D-s001

## Data Availability

The data supporting this article have been included as part of the ESI.†
